# Gut microbiota features associated with *Clostridioides difficile* colonization in puppies

**DOI:** 10.1371/journal.pone.0215497

**Published:** 2019-08-30

**Authors:** Alexander S. F. Berry, Brendan J. Kelly, Denise Barnhart, Donna J. Kelly, Daniel P. Beiting, Robert N. Baldassano, Laurel E. Redding

**Affiliations:** 1 Division of Gastroenterology, Hepatology, and Nutrition, Children’s Hospital of Philadelphia, Philadelphia, Pennsylvania, United States of America; 2 Department of Pathobiology, School of Veterinary Medicine, University of Pennsylvania, Philadelphia, Pennsylvania, United States of America; 3 Divisions of Infectious Diseases and Epidemiology, Perelman School of Medicine, University of Pennsylvania, Philadelphia, Pennsylvania, United States of America; 4 Department of Pathobiology, University of Pennsylvania, School of Veterinary Medicine, Kennett Square, Pennsylvania, United States of America; 5 Department of Clinical Sciences, University of Pennsylvania, School of Veterinary Medicine, Kennett Square, Pennsylvania, United States of America; University of Minnesota Twin Cities, UNITED STATES

## Abstract

In people, colonization with *Clostridioides difficile*, the leading cause of antibiotic-associated diarrhea, has been shown to be associated with distinct gut microbial features, including reduced bacterial community diversity and depletion of key taxa. In dogs, the gut microbiota features that define *C*. *difficile* colonization are less well understood. We sought to define the gut microbiota features associated with *C*. *difficile* colonization in puppies, a population where the prevalence of *C*. *difficile* has been shown to be elevated, and to define the effect of puppy age and litter upon these features and *C*. *difficile* risk. We collected fecal samples from weaned (n = 27) and unweaned (n = 74) puppies from 13 litters and analyzed the effects of colonization status, age and litter on microbial diversity using linear mixed effects models.

Colonization with *C*. *difficile* was significantly associated with younger age, and colonized puppies had significantly decreased bacterial community diversity and differentially abundant taxa compared to non-colonized puppies, even when adjusting for age. *C*. *difficile* colonization remained associated with decreased bacterial community diversity, but the association did not reach statistical significance in a mixed effects model incorporating litter as a random effect.

Even though litter explained a greater proportion (67%) of the variability in microbial diversity than colonization status, we nevertheless observed heterogeneity in gut microbial community diversity and colonization status within more than half of the litters, suggesting that the gut microbiota contributes to colonization resistance against *C*. *difficile*. The colonization of puppies with *C*. *difficile* has important implications for the potential zoonotic transfer of this organism to people. The identified associations point to mechanisms by which *C*. *difficile* colonization may be reduced.

## Introduction

*Clostridioides difficile* is a spore-forming anaerobic, gram-positive bacillus that is the leading cause of antibiotic-associated and nosocomial diarrhea in humans [1, 2] and a significant enteric pathogen in many species of animals [3–5]. Administration of antibiotics is the primary risk factor for the development of *C*. *difficile* infection (CDI) [1, 6, 7]. However, patients can develop CDI outside of a healthcare facility without the prior use of antibiotics, and community-acquired CDIs are now thought to account for one quarter of infections [8, 9].

The source of community-acquired infections has not been definitively established. People asymptomatically colonized with *C*. *difficile* are potential reservoirs [10], but zoonotic, environmental, and food-borne transmission to people has also been posited. The presence of *C*. *difficile* in companion animals has been documented since the 1980’s, and dogs and cats were posited as a potential reservoir species as early as 1983 [11]. Given the close contact between people and their pets, colonized or infected companion animals may represent an important transmission source for this pathogen. As in other species of animals [12–15], including human infants [16–18], *C*. *difficile* is highly prevalent in the feces of puppies [19–21]. Understanding how colonization is regulated in puppies might reduce their colonization with *C*. *difficile* and the potential transmission to pet owners.

The role of the commensal gut microbiota in *C*. *difficile* colonization resistance has been demonstrated in people [22–26] and in certain species of animals [27–29]. Human subject and animal model studies suggest key microbiome features, including community diversity and specific taxa, are involved in protection against *C*. *difficile*. No such association has been demonstrated in dogs, and studies of the association between the administration of antibiotics (and the consequent disruption of the gut microbiota) and *C*. *difficile* colonization/infection in dogs have yielded mixed results [30, 31]. The evolution of the neonatal canine gut microbiota has been described, with increasing diversity and taxonomic shifts occurring with increasing age [32]. As has been found in human infants [24], it is possible that certain taxonomic patterns and a lack of microbial community diversity in the gut may be associated with a lack of colonization resistance to *C*. *difficile*.

The objective of this study was to define the gut microbiota features associated with *C*. *difficile* colonization in puppies and to define the effects of puppy age and litter on the risk of colonization. The results could contribute to a better understanding of *C*. *difficile* colonization in puppies and their potential to serve as a reservoir for this pathogen.

## Materials and methods

### Samples

Freshly voided fecal samples were obtained from 1) pet owners bringing their puppies to the pediatric service at the Veterinary Hospital of the University of Pennsylvania, 2) shelters, and 3) breeders in the greater Philadelphia area who collected fecal samples from their puppies and shipped them in sterile conical screw-cap collection containers on ice overnight to the laboratory. No puppies were systemically ill at the time of sampling according to the pet owners, shelter workers or breeders who provided the samples, and none of the puppies had received antimicrobial therapy. After collection, samples were split into sterile cryogenic vials. One aliquot was processed for culture within 24 hours, while others were stored at -80°C and processed subsequently in batch for the 16S ribosomal RNA (rRNA) sequencing. Frozen samples were thawed only once prior to processing. This study was approved by the Institutional Animal Care and Use Committee of the University of Pennsylvania (protocol number 806539).

### Anaerobic culture and toxigenic testing

A 0.5 g pellet of formed fecal sample was mixed with 0.5 ml of 100% ethanol. The mixture remained for 60 minutes at room temperature before being inoculated on BBL CDSA/*Clostridioides difficile* selective agar (BD; Sparks, Maryland, USA) and Columbia CNA agar (Remel; Lenexa, KS, USA). Inoculated plates were incubated at 35°C under anaerobic growth conditions for seven days and checked for growth every other day. Suspect colonies were identified and isolated. Isolates were confirmed to be *C*. *difficile* by Maldi-TOF MS identification and/or RapID ANA II System (ThermoFisher Scientific, USA). Confirmed isolates of *C*. *difficile* were inoculated into BHI broth and/or cooked meat broth to induce toxin production. The broth was incubated anaerobically at 35°C for 48 hours. The supernatant was collected and tested by EIA (TechLab *C*. *difficile* Tox A/B II) for toxin production.

### 16 S sequencing

DNA was extracted from the fecal samples using Qiagen Power Soil DNA Extraction Kit (Qiagen, Hilden, Germany) using 0.25 g of each fecal pellet as input. Extraction and PCR blanks were used to control for environmental contamination and mock communities were used to control for contamination across wells. The V4 region of the 16S rRNA gene was amplified using barcoded primers for use on the Illumina platform [33]. The concentration of each PCR product was determined using a PicoGreen assay, and samples were normalized to equal amounts and pooled. Sequencing was performed using 250-base paired-end chemistry on an Illumina MiSeq instrument with an average read depth of 49,436 reads per sample. Three samples were dropped due to low read depth (<4000 reads per sample), raising the average read depth to 50,860 reads per sample. Sequences were demultiplexed using the Quantitative Insights into Microbial Ecology (QIIME2) software [34], and denoised using DADA2 [35]. Sequences were aligned using Maaft [36] and phylogenetic reconstruction was performed using Fasttree [37]. Finally, sequences were rarefied to 11,700 reads per sample for calculating alpha- and beta-diversity metrics.

### Analysis

The effects of age and litter on culture status were analyzed by logistic regression. Metrics of alpha and beta diversity of the fecal microbiota were calculated using the qiime diversity core-metrics-phylogenetic function in qiime2 and visualized using QIIME2 and Emperor [38].

The alpha diversity was calculated for each sample using the Shannon index. Differences in alpha diversity between *C*. *difficile*-infected and uninfected puppies were assessed using (1) univariable linear regression (N = 98), (2) linear regression controlling for puppy age (N = 98), and (3) a linear mixed effects model (LMM) on all unweaned puppies, controlling for age and using litter as a random effect (N = 70) using the lme4 package in R [39]. The effect of *C*. *difficile* colonization status on microbiota alpha diversity was assessed by comparing the likelihoods of the LMM with and without the fixed effect of *C*. *difficile* infection status using an analysis of variance. Finally, the effect of *C*. *difficile* toxigenicity on alpha diversity among *C*. *difficile*-positive puppies was assessed using univariable linear regression (N = 35).

The effect of *C*. *difficile* culture status on the per-specimen bacterial community diversity of the fecal microbiota was first assessed by univariable analysis. Univariable analysis was also performed to identify clustering of specimens by colonization status, using the PERMANOVA test applied to pairwise distances as determined by the beta diversity metrics Bray-Curtis, unweighted unifrac, and weighted unifrac. The effect of *C*. *difficile* culture status on beta diversity of the microbiota adjusted for puppy age and litter was assessed using mixed effects PERMANOVA. Age and culture status were considered fixed effects, while litter was considered a random effect. All comparisons were two-tailed, and P < 0.05 was considered to represent statistical significance. PERMANOVA tests were performed using the vegan package [40] as implemented in R v.3.5.2 (R Core Team, 2018). Principal coordinates analysis (PCoA) was performed using phyloseq [41] to visualize the clustering of samples by various parameters (*C*. *difficile* status, age, litter).

A taxonomic classifier trained on the GreenGenes database with 99% Operational taxonomic units (OTUs) was used to assign relative abundances of OTUs for each sample calculated at the genus level. The relative contributions of different microbial taxa that characterize the differences between *C*. *difficile* culture positive and negative puppies were assessed through linear discriminant analysis effect size (LEfSe) using the tools found at http://huttenhower.sph.harvard.edu/galaxy/. OTUs were filtered such that only those with >5% relative abundance in one or more samples and with LDA scores > 2.0 were considered to be significant. All plots were generated using the ggplot2 package in R [42].

## Results

### Subject characteristics and *C*. *difficile* status

**Fig 1:**
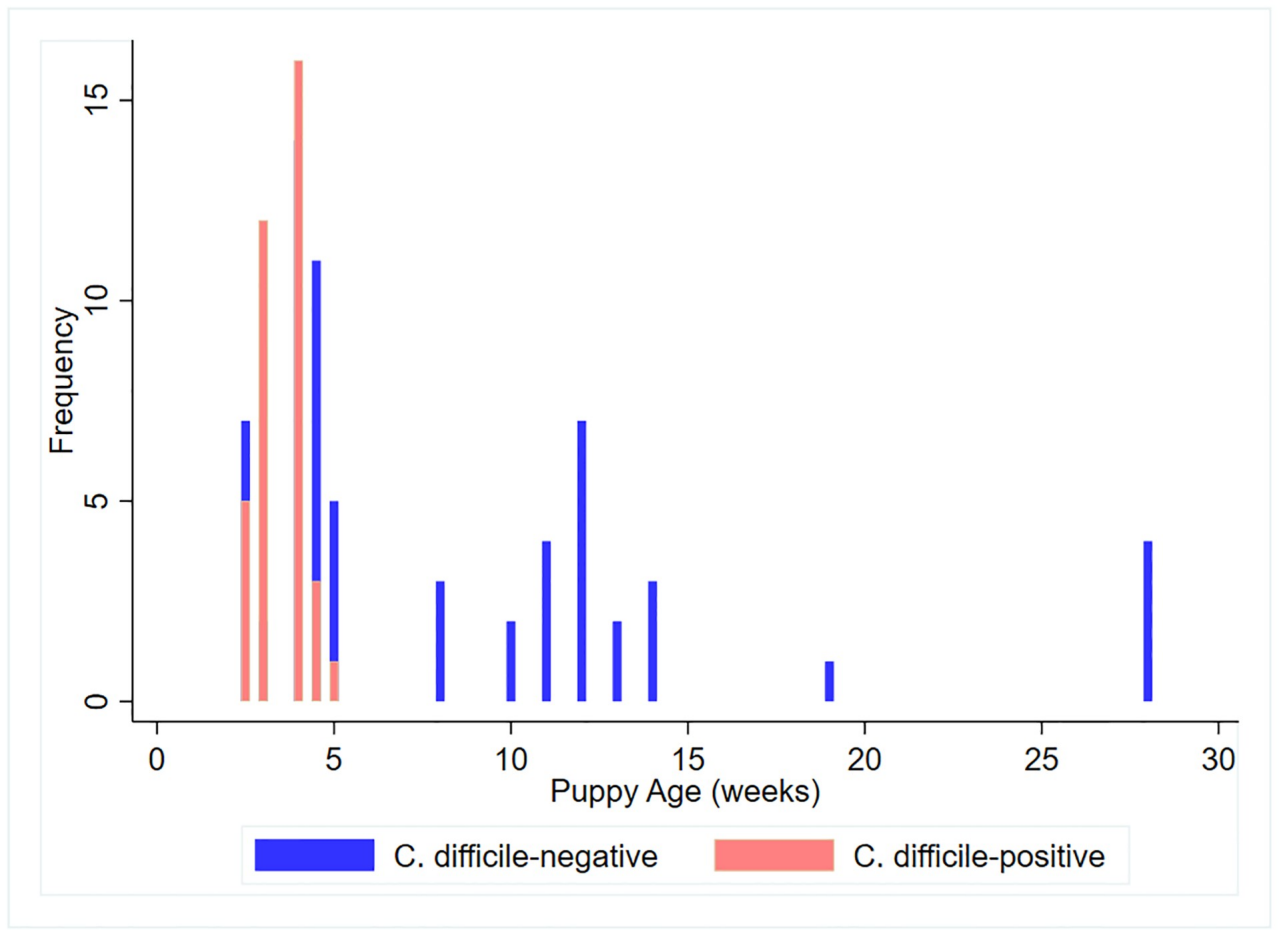
Distribution of the ages of puppies sampled in the greater Philadelphia region.

A total of 101 samples were collected from puppies ranging in age from 2–28 weeks. Seventy-four of the samples were obtained from 13 different litters of puppies that were still with their dam, and 27 samples were obtained from older weaned puppies that had been placed with families. The distribution of age was bi-modal, with the age of unweaned puppies in litters being significantly lower (p = 0.01) than that of the weaned puppies (Fig 1). The mean (SD) age of the unweaned puppies was 3.7 (0.8) weeks, whereas that of the weaned puppies was 11.4 (2.9) weeks. Litters ranged in size from 3 to 12 puppies, with a mean (SD) of 5.8 (2.9) puppies. The 13 litters included one litter each of Corgis, Golden Retrievers, Great Danes, Labrador Retrievers, French Bulldogs, Springer Spaniels, and Boerboels, two litters of Goldendoodles, and four litters of Collies. Most of the weaned puppies were mixed breed, although there was one Coton de Tulear, one Cocker Spaniel and one Corgi.

Thirty-seven samples (36.3%) were culture-positive for *C*. *difficile*, and 19 (51%) of these *C*. *difficile* isolates were toxigenic. All of samples from the weaned puppies (n = 27) were culture-negative. In 6 of the 13 litters of puppies, colonization status was the same for all puppies (i.e., all puppies within the litter were culture-negative or culture-positive). Age was significantly associated with culture status, with younger puppies being significantly more likely to harbor *C*. *difficile* (OR = 0.46, p = 0.004, 95% CI = 0.27–0.78).

### Association between *C*. *difficile* status, age and microbiota diversity in all puppies

**Fig 2:**
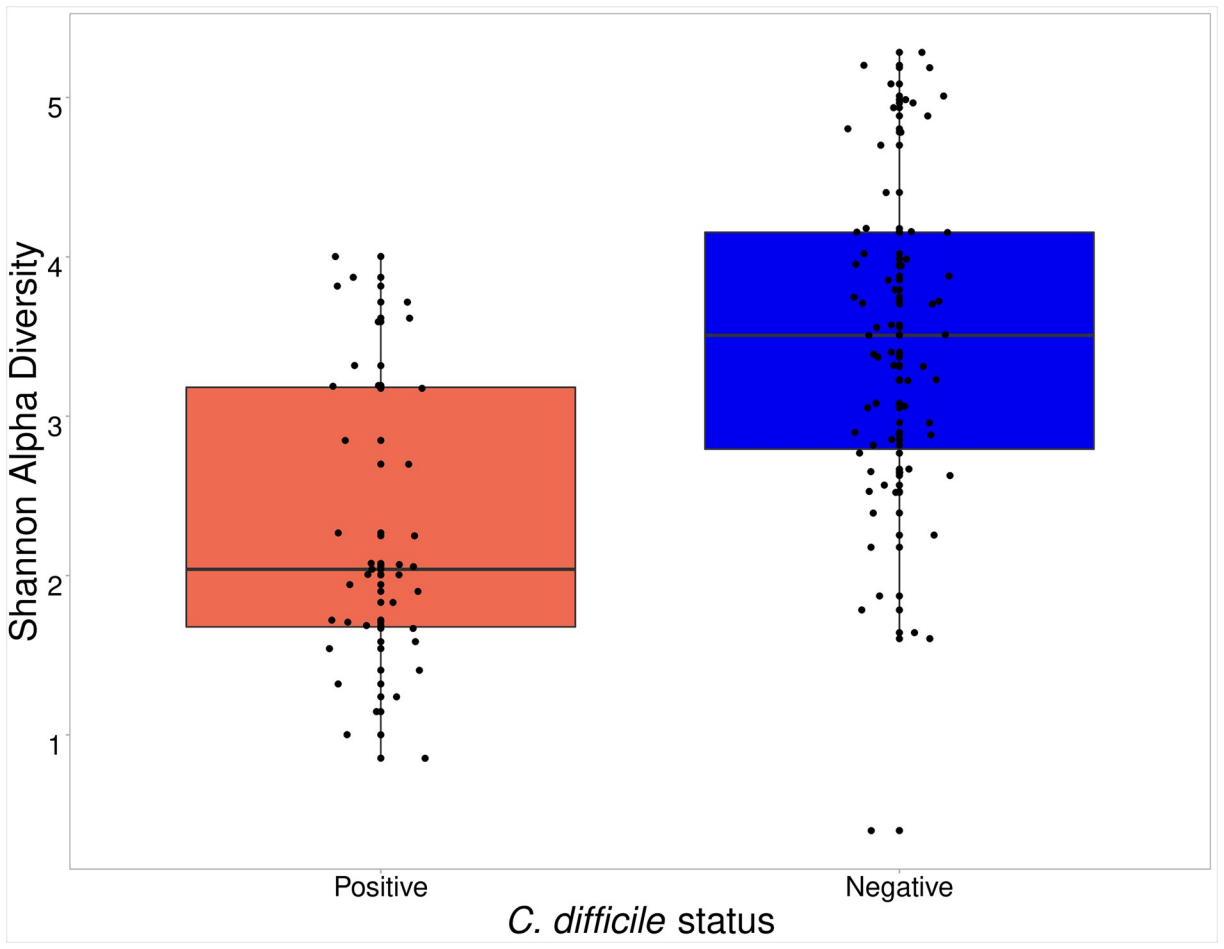
Boxplot of the Shannon diversity indices among *C*. *difficile*-positive puppies (left) and *C*. *difficile*-negative puppies (right). Boxes display the median, first and third quartiles, and whiskers extend to the minimum and maximum, while points represent outliers.

Microbiota community structure of 101 puppy fecal samples was assessed by sequencing and analyzing the V4 region of the 16S rRNA gene. Three culture-negative samples were dropped from subsequent analyses because of low coverage. Alpha diversity was significantly lower (p<0.001) in the *C*. *difficile*-positive fecal samples than in the *C*. *difficile*-negative fecal samples (Fig 2). When adjusting for age, the effect of *C*. *difficile* status on microbial community diversity was mitigated but persistent (p-value increased 2 orders of magnitude from 1.6 e^-7^ to 6e^-5^). There was no difference in diversity between puppies colonized with toxigenic *C*. *difficile* and non-toxigenic *C*. *difficile* (p = 0.66).

**Fig 3:**
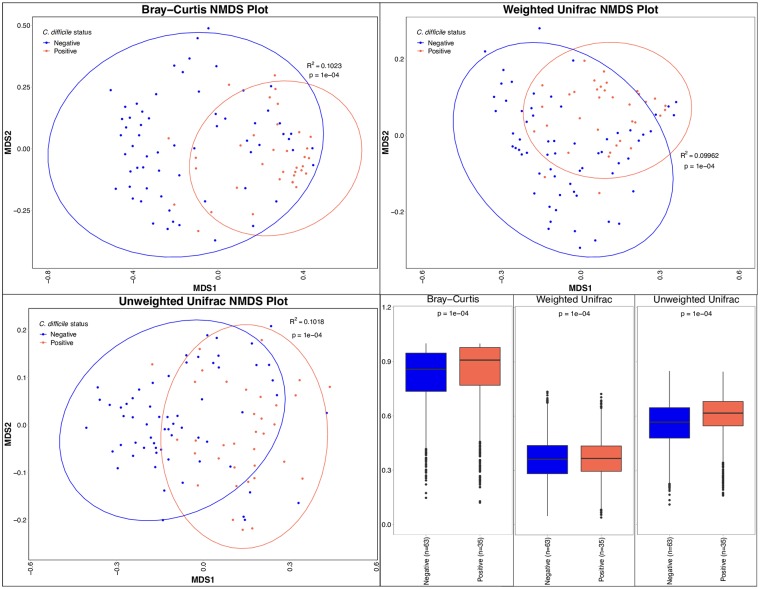
Non-metric Multidimensional Scaling (NMDS) plots and box plots show the dissimilarity in bacterial communities in *C*. *difficile*-positive and *C*. *difficile*-negative fecal samples from puppies in the greater Philadelphia area. Ellipses on NMDS plots display 95% confidence intervals. P-values were calculated using PERMANOVA. The dissimilarity among *C*. *difficile* positive puppies is displayed in the left boxplots and the dissimilarity between culture positive and negative puppies is displayed in the right boxplots. Boxes display the median, first and third quartiles, and whiskers extend to the minimum and maximum, while points represent outliers.

**Fig 4:**
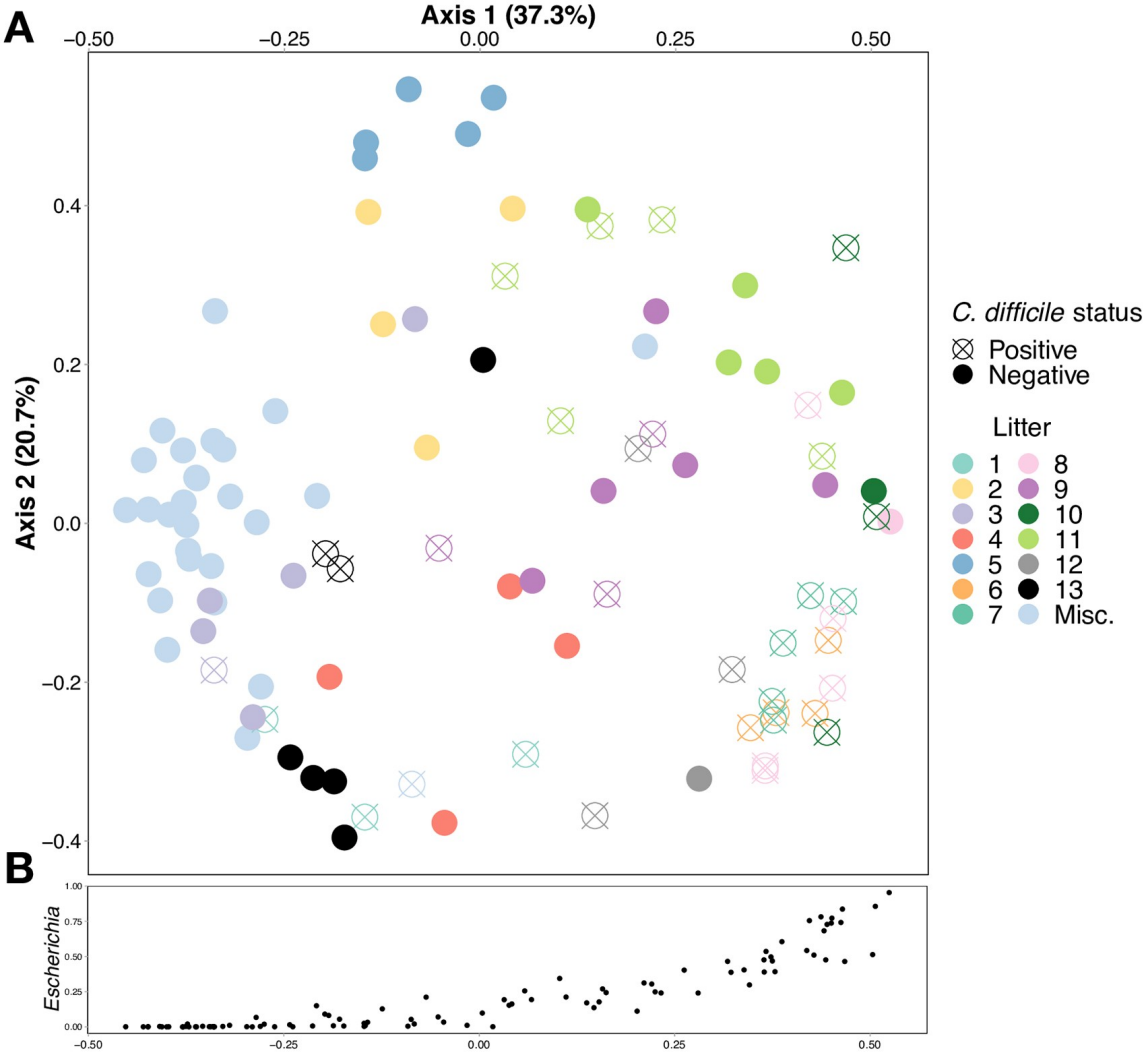
**A. Bray-Curtis principal coordinate analysis (PCoA) shows clustering of fecal samples from puppies in the greater Philadelphia area by *C*. *difficile* colonization status and by litter**. Fecal samples labeled “Misc.” are from older weaned puppies that were no longer in litters and resided with their owner. **B. Relative abundance of the genus *Escherichia* increases along the x-axis of the PCoA**.

Beta diversity, or the dissimilarity between microbiota communities, was assessed using Bray-Curtis, weighted unifrac, and unweighted unifrac. Univariable analysis showed a significant difference between microbial communities using all three metrics (p = 0.0001) even when controlling for age (p<0.0002) (Fig 3). The Bray-Curtis dissimilarity is summarized in a PCoA plot (Fig 4).

**Fig 5:**
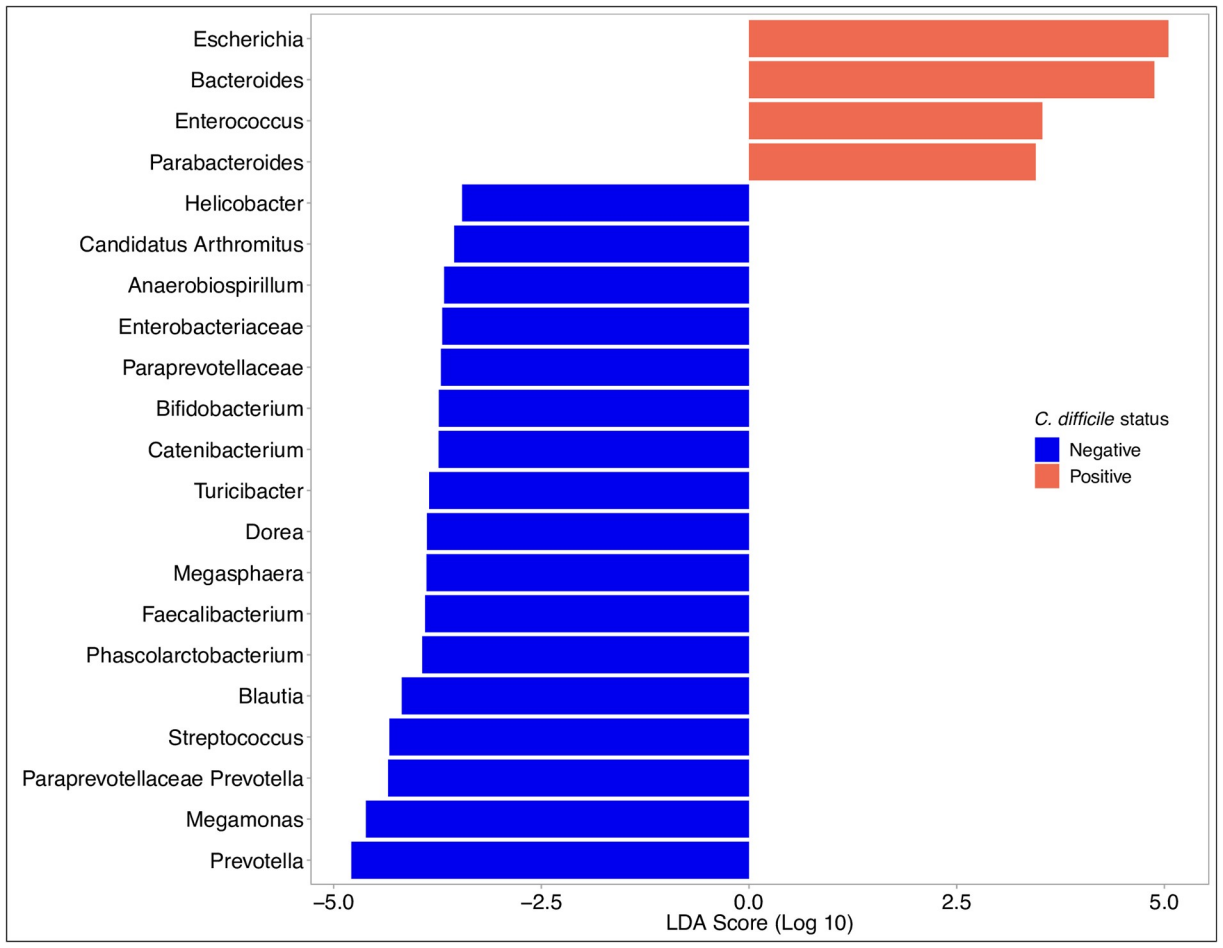
Linear discriminant analysis effect size analysis shows genera of bacteria that are differentially expressed in the *C*. *difficile*-positive and *C*. *difficile*-negative fecal samples from puppies in the greater Philadelphia area. Only organization taxonomic units with >5% relative abundance in one or more samples and with LDA scores > 2.0 are shown.

We found several taxa of bacteria to be differentially enriched in the *C*. *difficile*-positive and -negative samples. *C*. *difficile*-positive samples were enriched with members the *Escherichia*, *Bacteroides*, *Enterococcus* and *Parabacteroides* genera (Fig 5). Taxa from the *Escherichia* genus were found at relative abundance levels exceeding 10% in 48 samples and 50% in 15 samples. The relative abundance of *Escherichia* was associated with much of the clustering along the axis of principal component 1 (Fig 4, S1 Fig). In contrast, *C*. *difficile*-negative samples were enriched with members of the *Prevotella*, *Megamonas*, and *Streptococcus* genera. Unweaned puppies that were not colonized with *C*. *difficile* had higher relative abundance of taxa from the *Clostridia* genera than unweaned puppies that were colonized with *C*. *difficile*.

### Association between *C*. *difficile* status, age, litter, and microbiota diversity in unweaned puppies

To evaluate the effect of litter on the observed association between fecal bacterial community diversity and *C*. *difficile* colonization, we restricted analysis to the 70 unweaned puppies from 13 litters for which litter data were available. In seven litters, there was a mix of colonized and non-colonized puppies. (litters 3, 8, 9, 10, 11, 12, 13, Fig 5). In six litters, all of the puppies were of the same colonization status (all negative: litters 2,4,5; all positive: litters 1, 6, 7; Fig 5). When controlling for litter, *C*. *difficile* status had no effect on microbial alpha diversity (p = 0.547). Among these unweaned puppies, the litter explained most (67%, p = 1.0e-4) of the dissimilarity between bacterial communities, and colonization with *C*. *difficile* was no longer significantly correlated with microbiota composition (p > 0.1). PCoA analysis showed distinct clustering within most litters, but not necessarily by colonization status within a litter (S2 Fig). Even when controlling for breed, we found that litter remains a strong predictor of alpha and beta diversity. Among the 21 collies from four litters, Shannon alpha and beta diversity were significantly correlated with litter (p = 0.001 according to Kruskal-Wallis test and p = 0.001 according to PERMANOVA test, respectively).

## Discussion

Asymptomatic carriage of *C*. *difficile* is common in the young of many species, including humans [43], dogs [20], pigs [12, 44, 45], and cattle [46]. In people, colonization with *C*. *difficile* has been shown to be associated with altered gut microbial diversity [24, 26, 47–49], but no studies have examined this association in young dogs. In adult dogs, *C*. *difficile* colonization was associated with reduced gut bacterial species and diversity [50]. In puppies, we found that the association between lower bacterial community diversity and *C*. *difficile* colonization was statistically significant even when accounting for age, and certain bacterial taxa were preferentially associated with *C*. *difficile* colonization.

As has been found in other studies [21, 32], both colonization with *C*. *difficile* and reduced gut microbial diversity in puppies were significantly associated with young age. Similar associations have also been found in human studies [24, 26, 47, 51]. However, within litters, this association was no longer significant. Puppies of a same litter are exposed to the same environment, consume the same diet (i.e., dam’s milk), and are cophrophagic. It is therefore not surprising that similar gut microbial communities are seen among puppies of a litter, as we found and as was found in a previous study of 30 German Shepherd litters [52]. Microbial communities, presumably along with *C*. *difficile*, are likely shared among littermates. However, even within litters, we noted heterogeneity in the fecal microbiota (Fig 5) and in colonization status. In more than half of the litters (7/13), there were colonized and non-colonized puppies, suggesting that either our sample sizes were too small to detect a significant association between colonization status and microbial diversity, or other unmeasured factors were associated with colonization. The heterogeneity in the fecal microbiota within a litter may be analogous to the cage effect in mice studies [53, 54], where significant interindividual differences in intestinal microbiota were seen among mice within a cage, even though they were bred and raised in highly controlled similar conditions.

While the association between gut microbial diversity and *C*. *difficile* colonization status did not attain statistical significance within a litter, it is likely that features of the gut microbiota nevertheless contribute to the establishment and persistence of *C*. *difficile*. We found *C*. *difficile*-positive samples to be enriched with members of the *Escherichia*, *Bacteroides*, *Enterococcus* and *Parabacteroides* genera, and *C*. *difficile*-negative samples with members of the *Prevotella*, *Megamonas*, and *Streptococcus* genera. Almost identical trends were found for taxa of the *Escherichia*, *Parabacteroides*, *Enterococcus*, *Prevotella* and *Megamonas* genera in one study comparing *C*. *difficile* non-colonized, asymptomatically colonized and infected human adults [26], and for taxa of the *Parabacteroides*, *Prevotella*, *Paraprevotella* and *Enterococcus* genera in another study of non-colonized and colonized human adults [49]. Similar findings were found for the *Bacteroides* genera in a study of human infants [51]. In particular, increased relative abundances of taxa from the *Parabacteroides* and *Enterococcus* genera are thought to be the result of a blooming phenomenon associated with reduced ecological niche competition in people with CDI [49, 55, 56]. In adult dogs, at the phylum level, *C*. *difficile* colonization was associated with increases in the relative abundance of Fusobacteria, Proteobacteria, and Firmicutes, and decreases in Verrucomicrobia, Bacteroidetes, Euryarchaeota, and Actinobacteria [50]. This is consistent with our finding of increased relative abundance of *Escherichia* and *Enterococcus* and decreased relative abundance of *Prevotella*, but inconsistent with our other findings.

Among unweaned puppies, we found that noncolonized puppies had higher relative abundances of taxa from the *Clostridia* genera compared to colonized puppies. Consistent with this finding, other studies have postulated that bacterial species that are phylogenetically related to *C*. *difficile* and share niches and compete for similar resources could provide colonization resistance against toxigenic *C*. *difficile* [57, 58]. In fact, colonization with non-toxigenic *C*. *difficile* has been shown to prevent infection with toxigenic *C*. *difficile* in hamsters and people following administration of antibiotics [58–60].

In contrast to our findings, one study showed that noncolonized human infants had lower relative abundance of taxa from the *Escherichia* genera than colonized infants [24], while several other studies found *Bacteroides* spp in greater relative abundance in non-colonized human infants, children and adults [26, 47, 61, 62]. It is unclear why these discrepancies were observed in our study. Both *Bacteroides* spp, which are used as markers of a healthy gut in people [62], and *E*. *coli* are found in the feces of healthy puppies [32, 63]. *Bacteroides* spp are found in increasing relative abundance with increasing age, while *E*. *coli* levels are significantly higher in younger (less than 21 days) puppies than in older (greater than 42 days) puppies [32]. Our findings underscore that puppies colonized with *C*. *difficile* retain microbial gut features consistent with those of healthy animals. Additionally, while depletion and enrichment of certain bacterial taxa in colonized puppies were mostly consistent with what is seen in people, certain distinctions point to possible species-specific interactions between various bacterial taxa and *C*. *difficile* in the gut microbiota.

While some of the general trends were similar in our study and in several human studies, it is important to note that GI microbiota differ significantly by species, and extrapolation from human to animals is not always possible or prudent. In one study, for example, microbial groups associated with *C*. *difficile* colonization status were significantly different for people and poultry [64]. However, the canine gut microbiome has been shown to be more similar to the human gut microbiome than that of pigs and mice [65, 66], perhaps due to their shared environments and diets, which might be why we observed similar microbiological trends in puppies and people.

The large proportion of puppies colonized with *C*. *difficile* has important implications for the potential zoonotic transmission of this organism. While it is likely that a puppy’s litter (and resultant environmental exposures) is the main determinant of colonization status, it is also likely that the puppy’s microbiota has an effect. The small number of puppies in each litter and the limited number of litters with colonized and non-colonized puppies precluded us from establishing whether the effect was statistically significant, but microbial community signatures that were consistent with what has been observed in people suggest that the microbiota has a role to play in colonization resistance. The protective role of the gut microbiota is particularly important when considering the fact that many puppies sold in pet stores (up to 95%) receive prophylactic antibiotics prior to shipping, as was recognized in a recent outbreak of Campylobacteriosis associated with puppies in pet stores [67]. This could result in gastrointestinal dysbiosis and a resultant predisposition to harboring pathogens such as *C*. *difficile*. More research is needed to better understand the interaction between the gut microbiota and colonization and infection with *C*. *difficile* in dogs, especially at the level of the litter; define the relationship between dog-colonizing *C*. *difficile* strains and human colonizing strains; and understand how interventions that reduce colonization in human pets may impact human disease prevention.

Our study had several limitations. The first is the possibility of false-negative culture results. *C*. *difficile* is difficult to isolate and is very sensitive to even low levels of oxygen in the environment [68], and other authors [69] have found recovery rates to be higher when using a broth enrichment step, which we did not perform. Second, we did not have information on puppies’ sex, husbandry status, environmental exposures (including contact with other animals, children, etc.), or dietary status, all of which can impact the gut microbiome. Finally, the cross-sectional nature of the study precludes the possibility of drawing any conclusions about the duration of colonization.

## Conclusions

We found that puppies with *C*. *difficile*-positive fecal samples had reduced gut microbiota diversity, even when adjusting for the puppy’s age, and that there were differentially-abundant taxa in *C*. *difficile*-positive and *C*. *difficile*-negative fecal samples. These differences in microbial features may be permissive in promoting the colonization and establishment of *C*. *difficile*, though longitudinal studies are needed to confirm this hypothesis. Though this effect was not observed at the level of the litter, and even though the litter explained a large proportion of the gut microbiota diversity, heterogeneity in the gut microbiota and in *C*. *difficile* colonization within litters was observed in more than half of the litters, suggesting that the gut microbiota and potentially other unmeasured factors contribute to colonization resistance against *C*. *difficile* in puppies.

## Supporting information

S1 Fig Principal component analysis (PCA) biplot shows the genera that drive the differences in microbiome community structure among A) all weaned and unweaned puppies (n = 98) and B) only unweaned puppies (n = 70). The PCoA was calculated using the prcomp function and visualized using the biplot function in R.
(TIF)

S2 Fig Principal coordinate analysis (PCoA) plot showing clustering of fecal samples from seven litters of puppies in the greater Philadelphia area where puppies within litters had different *C*. *difficile* colonization status. (TIF)

## References

[1] Lessa FC, Winston LG, McDonald LC, Emerging Infections Program CdST. Burden of Clostridium difficile infection in the United States. The New England journal of medicine. 2015;372(24):2369–70. 26061850 10.1056/NEJMc1505190 PMC10880113

[2] Smits WK, Lyras D, Lacy DB, Wilcox MH, Kuijper EJ. Clostridium difficile infection. Nature reviews Disease primers. 2016;2:16020 10.1038/nrdp.2016.20 27158839 PMC5453186

[3] Keessen EC, Gaastra W, Lipman LJ. Clostridium difficile infection in humans and animals, differences and similarities. Veterinary microbiology. 2011;153(3–4):205–17. 10.1016/j.vetmic.2011.03.020 21530110

[4] Moono P, Foster NF, Hampson DJ, Knight DR, Bloomfield LE, Riley TV. Clostridium difficile Infection in Production Animals and Avian Species: A Review. Foodborne pathogens and disease. 2016;13(12):647–55. 10.1089/fpd.2016.2181 27602596

[5] Songer JG, Anderson MA. Clostridium difficile: an important pathogen of food animals. Anaerobe. 2006;12(1):1–4. 10.1016/j.anaerobe.2005.09.001 16701605

[6] Thornton CS, Rubin JE, Greninger AL, Peirano G, Chiu CY, Pillai DR. Epidemiological and genomic characterization of community-acquired Clostridium difficile infections. BMC Infect Dis. 2018;18(1):443 10.1186/s12879-018-3337-9 30170546 PMC6119286

[7] Tamma PD, Sandora TJ. Clostridium difficile Infection in Children: Current State and Unanswered Questions. J Pediatric Infect Dis Soc. 2012;1(3):230–43. 10.1093/jpids/pis071 23687578 PMC3656539

[8] Khanna S, Baddour LM, Huskins WC, Kammer PP, Faubion WA, Zinsmeister AR, et al The epidemiology of Clostridium difficile infection in children: a population-based study. Clin Infect Dis. 2013;56(10):1401–6. 10.1093/cid/cit075 23408679 PMC3693491

[9] Karlstrom O, Fryklund B, Tullus K, Burman LG. A prospective nationwide study of Clostridium difficile-associated diarrhea in Sweden. The Swedish C. difficile Study Group. Clin Infect Dis. 1998;26(1):141–5. 10.1086/516277 9455523

[10] Galdys AL, Curry SR, Harrison LH. Asymptomatic Clostridium difficile colonization as a reservoir for Clostridium difficile infection. Expert review of anti-infective therapy. 2014;12(8):967–80. 10.1586/14787210.2014.920252 24848084

[11] Borriello SP, Honour P, Turner T, Barclay F. Household pets as a potential reservoir for Clostridium difficile infection. J Clin Pathol. 1983;36(1):84–7. 10.1136/jcp.36.1.84 6822681 PMC498110

[12] Hopman NE, Keessen EC, Harmanus C, Sanders IM, van Leengoed LA, Kuijper EJ, et al Acquisition of Clostridium difficile by piglets. Veterinary microbiology. 2011;149(1–2):186–92. 10.1016/j.vetmic.2010.10.013 21111541

[13] Hammitt MC, Bueschel DM, Keel MK, Glock RD, Cuneo P, DeYoung DW, et al A possible role for Clostridium difficile in the etiology of calf enteritis. Veterinary microbiology. 2008;127(3–4):343–52. 10.1016/j.vetmic.2007.09.002 17964088 PMC7131641

[14] Jones RL, Adney WS, Alexander AF, Shideler RK, Traub-Dargatz JL. Hemorrhagic necrotizing enterocolitis associated with Clostridium difficile infection in four foals. J Am Vet Med Assoc. 1988;193(1):76–9. 3262102

[15] Baverud V, Gustafsson A, Franklin A, Aspan A, Gunnarsson A. Clostridium difficile: prevalence in horses and environment, and antimicrobial susceptibility. Equine veterinary journal. 2003;35(5):465–71. 12875324 10.2746/042516403775600505

[16] Collignon A, Ticchi L, Depitre C, Gaudelus J, Delmee M, Corthier G. Heterogeneity of Clostridium difficile isolates from infants. European journal of pediatrics. 1993;152(4):319–22. 10.1007/bf01956743 8482281

[17] Rousseau C, Poilane I, De Pontual L, Maherault AC, Le Monnier A, Collignon A. Clostridium difficile carriage in healthy infants in the community: a potential reservoir for pathogenic strains. Clin Infect Dis. 2012;55(9):1209–15. 10.1093/cid/cis637 22843784

[18] Adlerberth I, Huang H, Lindberg E, Aberg N, Hesselmar B, Saalman R, et al Toxin-producing Clostridium difficile strains as long-term gut colonizers in healthy infants. Journal of clinical microbiology. 2014;52(1):173–9. 10.1128/JCM.01701-13 24172156 PMC3911410

[19] Buogo C, Burnens AP, Perrin J, Nicolet J. [Presence of Campylobacter spp., Clostridium difficile, C. perfringens and salmonellae in litters of puppies and in adult dogs in a shelter]. Schweizer Archiv fur Tierheilkunde. 1995;137(5):165–71. 7569838

[20] Perrin J, Buogo C, Gallusser A, Burnens AP, Nicolet J. Intestinal carriage of Clostridium difficile in neonate dogs. Zentralblatt fur Veterinarmedizin Reihe B Journal of veterinary medicine Series B. 1993;40(3):222–6. 10.1111/j.1439-0450.1993.tb00131.x 8342371

[21] Alvarez-Perez S, Blanco JL, Pelaez T, Lanzarot MP, Harmanus C, Kuijper E, et al Faecal shedding of antimicrobial-resistant Clostridium difficile strains by dogs. The Journal of small animal practice. 2015;56(3):190–5. 10.1111/jsap.12311 25483272

[22] Britton RA, Young VB. Interaction between the intestinal microbiota and host in Clostridium difficile colonization resistance. Trends Microbiol. 2012;20(7):313–9. 10.1016/j.tim.2012.04.001 22595318 PMC3408078

[23] Chang JY, Antonopoulos DA, Kalra A, Tonelli A, Khalife WT, Schmidt TM, et al Decreased diversity of the fecal Microbiome in recurrent Clostridium difficile-associated diarrhea. J Infect Dis. 2008;197(3):435–8. 10.1086/525047 18199029

[24] Rousseau C, Levenez F, Fouqueray C, Dore J, Collignon A, Lepage P. Clostridium difficile colonization in early infancy is accompanied by changes in intestinal microbiota composition. Journal of clinical microbiology. 2011;49(3):858–65. 10.1128/JCM.01507-10 21177896 PMC3067754

[25] Samarkos M, Mastrogianni E, Kampouropoulou O. The role of gut microbiota in Clostridium difficile infection. Eur J Intern Med. 2018;50:28–32. 10.1016/j.ejim.2018.02.006 29428498

[26] Zhang L, Dong D, Jiang C, Li Z, Wang X, Peng Y. Insight into alteration of gut microbiota in Clostridium difficile infection and asymptomatic C. difficile colonization. Anaerobe. 2015;34:1–7. 10.1016/j.anaerobe.2015.03.008 25817005

[27] Burdet C, Sayah-Jeanne S, Nguyen TT, Hugon P, Sablier-Gallis F, Saint-Lu N, et al Antibiotic-Induced Dysbiosis Predicts Mortality in an Animal Model of Clostridium difficile Infection. Antimicrobial agents and chemotherapy. 2018;62(10). 10.1128/AAC.00925-18 PMC6153837 30061286

[28] Rodriguez C, Taminiau B, Brevers B, Avesani V, Van Broeck J, Leroux A, et al Faecal microbiota characterisation of horses using 16 rdna barcoded pyrosequencing, and carriage rate of clostridium difficile at hospital admission. BMC microbiology. 2015;15:181 10.1186/s12866-015-0514-5 26377067 PMC4573688

[29] Grzeskowiak LM, Pieper R, Huynh HA, Cutting SM, Vahjen W, Zentek J. Impact of early-life events on the susceptibility to Clostridium difficile colonisation and infection in the offspring of the pig. Gut Microbes. 2018:1–9. 10.1080/19490976.2018.1518554 PMC6546313 30252612

[30] Hussain I, Sharma RK, Borah P, Rajkhowa S, Hussain I, Barkalita LM, et al Isolation and characterization of Clostridium difficile from pet dogs in Assam, India. Anaerobe. 2015;36:9–13. 10.1016/j.anaerobe.2015.09.006 26393292

[31] Weese JS, Armstrong J. Outbreak of Clostridium difficile-associated disease in a small animal veterinary teaching hospital. Journal of veterinary internal medicine. 2003;17(6):813–6. 10.1111/j.1939-1676.2003.tb02519.x 14658717 PMC7202293

[32] Guard BC, Mila H, Steiner JM, Mariani C, Suchodolski JS, Chastant-Maillard S. Characterization of the fecal microbiome during neonatal and early pediatric development in puppies. PloS one. 2017;12(4):e0175718 10.1371/journal.pone.0175718 28448583 PMC5407640

[33] Jasarevic E, Howard CD, Morrison K, Misic A, Weinkopff T, Scott P, et al The maternal vaginal microbiome partially mediates the effects of prenatal stress on offspring gut and hypothalamus. Nat Neurosci. 2018;21(8):1061–71. 10.1038/s41593-018-0182-5 29988069

[34] Caporaso JG, Kuczynski J, Stombaugh J, Bittinger K, Bushman FD, Costello EK, et al QIIME allows analysis of high-throughput community sequencing data. Nature methods. 2010;7(5):335–6. 10.1038/nmeth.f.303 20383131 PMC3156573

[35] Callahan BJ, McMurdie PJ, Rosen MJ, Han AW, Johnson AJ, Holmes SP. DADA2: High-resolution sample inference from Illumina amplicon data. Nature methods. 2016;13(7):581–3. 10.1038/nmeth.3869 27214047 PMC4927377

[36] Katoh K, Standley DM. MAFFT multiple sequence alignment software version 7: improvements in performance and usability. Molecular biology and evolution. 2013;30(4):772–80. 10.1093/molbev/mst010 23329690 PMC3603318

[37] Price MN, Dehal PS, Arkin AP. FastTree 2—approximately maximum-likelihood trees for large alignments. PloS one. 2010;5(3):e9490 10.1371/journal.pone.0009490 20224823 PMC2835736

[38] Vazquez-Baeza Y, Pirrung M, Gonzalez A, Knight R. EMPeror: a tool for visualizing high-throughput microbial community data. Gigascience. 2013;2(1):16 10.1186/2047-217X-2-16 24280061 PMC4076506

[39] Bates D, Maechler M, Bolker B, Walker S. Fitting Linear Mixed-Effects Models Using lme4. Journal of Statistical Software. 2015;67(1):1–48.

[40] Oksanen J, Blanchet F, Kindt R. Package, "vegan". 2015.

[41] McMurdie PJ, Holmes S. phyloseq: an R package for reproducible interactive analysis and graphics of microbiome census data. PloS one. 2013;8(4):e61217 10.1371/journal.pone.0061217 23630581 PMC3632530

[42] Wickham H. ggplot2: Elegant Graphics for Data Analysis. New York, NY: Springer-Verlag; 2016.

[43] Kuijper EJ, Coignard B, Tull P, difficile ESGfC, States EUM, European Centre for Disease P, et al Emergence of Clostridium difficile-associated disease in North America and Europe. Clinical microbiology and infection: the official publication of the European Society of Clinical Microbiology and Infectious Diseases. 2006;12 Suppl 6:2–18. 10.1111/j.1469-0691.2006.01580.x 16965399

[44] Moono P, Putsathit P, Knight DR, Squire MM, Hampson DJ, Foster NF, et al Persistence of Clostridium difficile RT 237 infection in a Western Australian piggery. Anaerobe. 2016;37:62–6. 10.1016/j.anaerobe.2015.11.012 26679487

[45] Weese JS, Wakeford T, Reid-Smith R, Rousseau J, Friendship R. Longitudinal investigation of Clostridium difficile shedding in piglets. Anaerobe. 2010;16(5):501–4. 10.1016/j.anaerobe.2010.08.001 20708700

[46] Knight DR, Thean S, Putsathit P, Fenwick S, Riley TV. Cross-sectional study reveals high prevalence of Clostridium difficile non-PCR ribotype 078 strains in Australian veal calves at slaughter. Applied and environmental microbiology. 2013;79(8):2630–5. 10.1128/AEM.03951-12 23396338 PMC3623178

[47] Rea MC, O’Sullivan O, Shanahan F, O’Toole PW, Stanton C, Ross RP, et al Clostridium difficile carriage in elderly subjects and associated changes in the intestinal microbiota. Journal of clinical microbiology. 2012;50(3):867–75. 10.1128/JCM.05176-11 22162545 PMC3295116

[48] Chen LA, Hourigan SK, Grigoryan Z, Gao Z, Clemente JC, Rideout JR, et al Decreased Fecal Bacterial Diversity and Altered Microbiome in Children Colonized With Clostridium difficile. Journal of pediatric gastroenterology and nutrition. 2018. 10.1097/MPG.0000000000002210 30540709

[49] Han SH, Yi J, Kim JH, Lee S, Moon HW. Composition of gut microbiota in patients with toxigenic Clostridioides (Clostridium) difficile: Comparison between subgroups according to clinical criteria and toxin gene load. PloS one. 2019;14(2):e0212626 10.1371/journal.pone.0212626 30785932 PMC6382146

[50] Stone NE, Nunnally AE, Jimenez V Jr., Cope EK, Sahl JW, Sheridan K, et al Domestic canines do not display evidence of gut microbial dysbiosis in the presence of Clostridioides (Clostridium) difficile, despite cellular susceptibility to its toxins. Anaerobe. 2019 10.1016/j.anaerobe.2019.03.017 . 30946985

[51] Fallani M, Rigottier-Gois L, Aguilera M, Bridonneau C, Collignon A, Edwards CA, et al Clostridium difficile and Clostridium perfringens species detected in infant faecal microbiota using 16S rRNA targeted probes. J Microbiol Methods. 2006;67(1):150–61. 10.1016/j.mimet.2006.03.010 16647148

[52] Vilson A, Ramadan Z, Li Q, Hedhammar A, Reynolds A, Spears J, et al Disentangling factors that shape the gut microbiota in German Shepherd dogs. PloS one. 2018;13(3):e0193507 10.1371/journal.pone.0193507 29570709 PMC5865712

[53] McCafferty J, Muhlbauer M, Gharaibeh RZ, Arthur JC, Perez-Chanona E, Sha W, et al Stochastic changes over time and not founder effects drive cage effects in microbial community assembly in a mouse model. The ISME journal. 2013;7(11):2116–25. 10.1038/ismej.2013.106 23823492 PMC3806260

[54] Hoy YE, Bik EM, Lawley TD, Holmes SP, Monack DM, Theriot JA, et al Variation in Taxonomic Composition of the Fecal Microbiota in an Inbred Mouse Strain across Individuals and Time. PloS one. 2015;10(11):e0142825 10.1371/journal.pone.0142825 26565698 PMC4643986

[55] Milani C, Ticinesi A, Gerritsen J, Nouvenne A, Lugli GA, Mancabelli L, et al Gut microbiota composition and Clostridium difficile infection in hospitalized elderly individuals: a metagenomic study. Scientific reports. 2016;6:25945 10.1038/srep25945 27166072 PMC4863157

[56] Stecher B, Maier L, Hardt WD. ‘Blooming’ in the gut: how dysbiosis might contribute to pathogen evolution. Nature reviews Microbiology. 2013;11(4):277–84. 10.1038/nrmicro2989 23474681

[57] Perez-Cobas AE, Moya A, Gosalbes MJ, Latorre A. Colonization Resistance of the Gut Microbiota against Clostridium difficile. Antibiotics (Basel). 2015;4(3):337–57. 27025628 10.3390/antibiotics4030337 PMC4790290

[58] Shim JK, Johnson S, Samore MH, Bliss DZ, Gerding DN. Primary symptomless colonisation by Clostridium difficile and decreased risk of subsequent diarrhoea. Lancet. 1998;351(9103):633–6. 10.1016/S0140-6736(97)08062-8 9500319

[59] Merrigan MM, Sambol SP, Johnson S, Gerding DN. New approach to the management of Clostridium difficile infection: colonisation with non-toxigenic C. difficile during daily ampicillin or ceftriaxone administration. Int J Antimicrob Agents. 2009;33 Suppl 1:S46–50. 19303570 10.1016/S0924-8579(09)70017-2

[60] Kyne L, Warny M, Qamar A, Kelly CP. Asymptomatic carriage of Clostridium difficile and serum levels of IgG antibody against toxin A. The New England journal of medicine. 2000;342(6):390–7. 10.1056/NEJM200002103420604 10666429

[61] Hopkins MJ, Macfarlane GT. Changes in predominant bacterial populations in human faeces with age and with Clostridium difficile infection. Journal of medical microbiology. 2002;51(5):448–54. 10.1099/0022-1317-51-5-448 11990498

[62] Louie TJ, Emery J, Krulicki W, Byrne B, Mah M. OPT-80 eliminates Clostridium difficile and is sparing of bacteroides species during treatment of C. difficile infection. Antimicrobial agents and chemotherapy. 2009;53(1):261–3. 10.1128/AAC.01443-07 18955523 PMC2612159

[63] Smith HW. The development of the flora of the alimentary tract in young animals. J Pathol Bacteriol. 1965;90(2):495–513. 4285022

[64] Skraban J, Dzeroski S, Zenko B, Tusar L, Rupnik M. Changes of poultry faecal microbiota associated with Clostridium difficile colonisation. Vet Microbiol. 2013 8 30;165(3–4):416–24 10.1016/j.vetmic.2013.04.014 23664184

[65] Coelho LP, Kultima JR, Costea PI, Fournier C, Pan Y, Czarnecki-Maulden G, et al Similarity of the dog and human gut microbiomes in gene content and response to diet. Microbiome. 2018;6(1):72 10.1186/s40168-018-0450-3 29669589 PMC5907387

[66] Deng P, Swanson KS. Gut microbiota of humans, dogs and cats: current knowledge and future opportunities and challenges. Br J Nutr. 2015;113 Suppl:S6–17. 25414978 10.1017/S0007114514002943

[67] Montgomery M, Robertson S, Koski L, al. e. Multidrug-Resistant Campylobacter jejuni Outbreak Linked to Puppy Exposure—United States, 2016–2018. 2018. 10.15585/mmwr.mm6737a3 PMC6147421 30235182

[68] Edwards AN, Suarez JM, McBride SM. Culturing and maintaining Clostridium difficile in an anaerobic environment. J Vis Exp. 2013(79):e50787 10.3791/50787 24084491 PMC3871928

[69] Arroyo LG, Rousseau J, Willey BM, Low DE, Staempfli H, McGeer A, et al Use of a selective enrichment broth to recover Clostridium difficile from stool swabs stored under different conditions. Journal of clinical microbiology. 2005;43(10):5341–3. 10.1128/JCM.43.10.5341-5343.2005 16208013 PMC1248507

