# Supplementary figures and images for: Gut microbiota features associated with Clostridioides difficile colonization in puppies
Source: PLoS One. 2019 Aug 30;14(8):e0215497. doi: 10.1371/journal.pone.0215497 (PMC6716646; doi:10.1371/journal.pone.0215497)

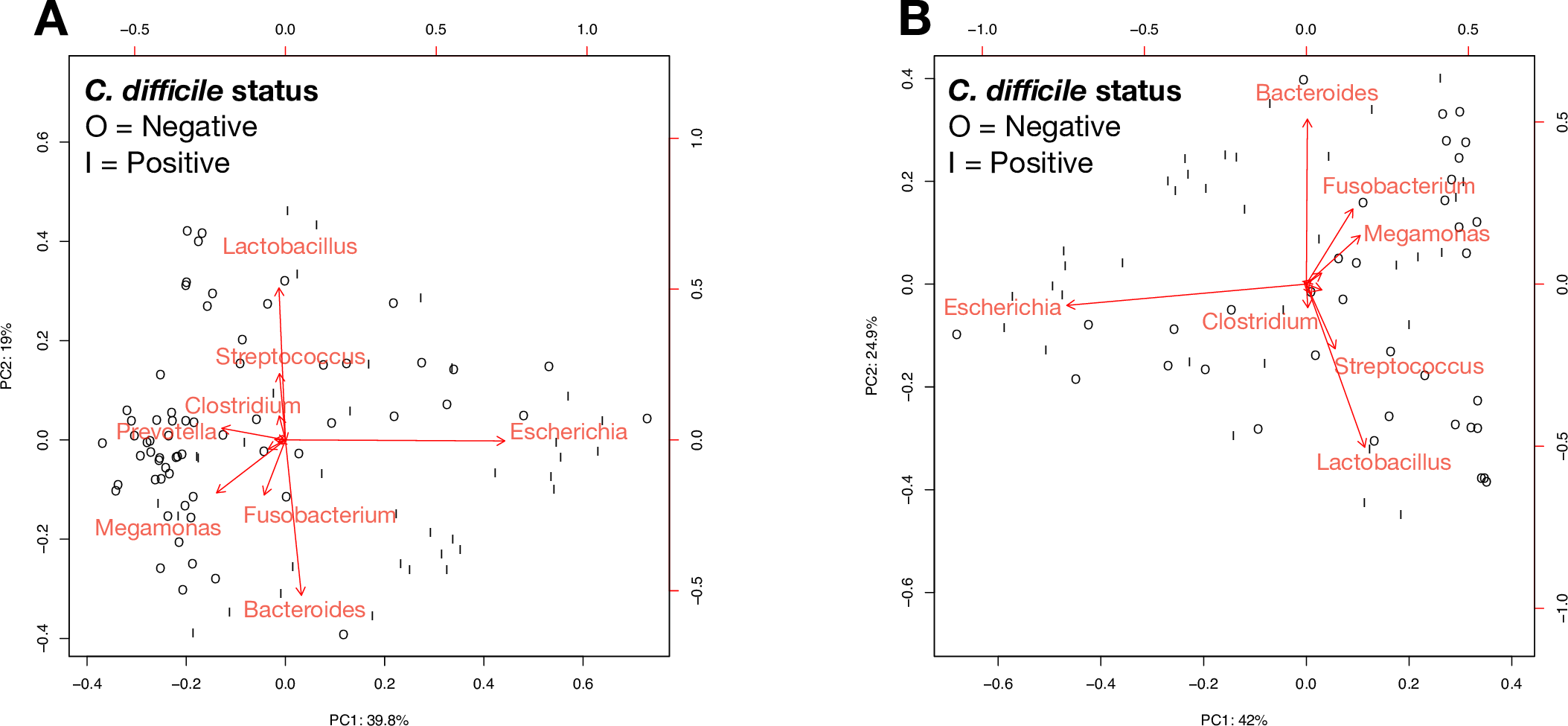

Supplement: S1 Fig — The PCoA was calculated using the prcomp function and visualized using the biplot function in R. (TIF) [file pone.0215497.s001.tif]

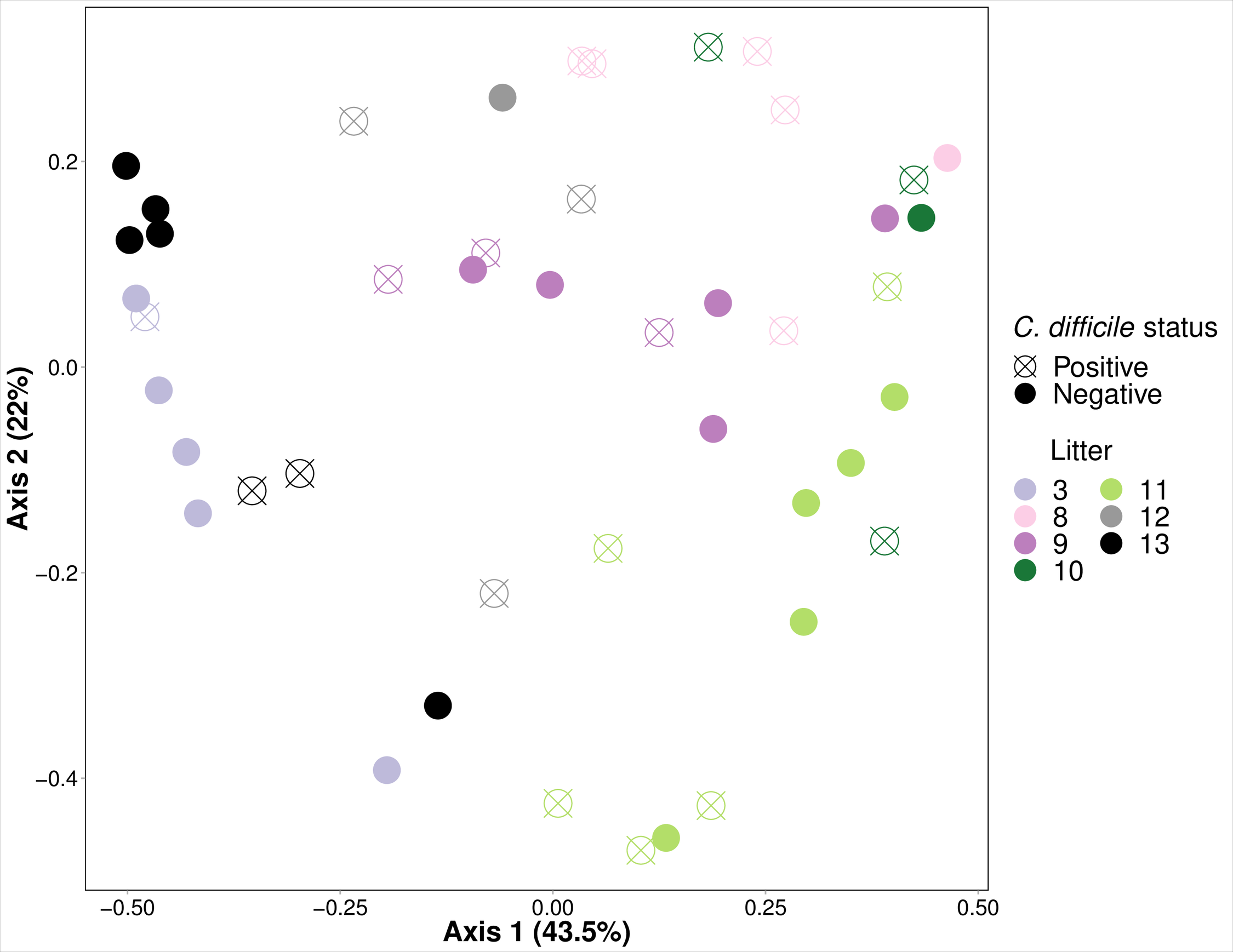

Supplement: S2 Fig — (TIF) [file pone.0215497.s002.tif]
